# Poly[diaqua­[μ_6_-4,4′-(1,4-phenyl­ene)bis­(2,6-dimethyl­pyridine-3,5-dicarboxyl­ato)]dilead(II)]

**DOI:** 10.1107/S1600536813007733

**Published:** 2013-03-28

**Authors:** Yi Zhu, Ming-Xing Zhang, Shan-Shan Yang, Feng Xiao, Xiao-Ping Zhang, Yuan-Yuan Gao, Bing-Jie Li, Kun-Lin Huang

**Affiliations:** a College of Life Science, and College of Chemistry, Chongqing Normal University, Chongqing 400047, People’s Republic of China

## Abstract

The asymmetric unit of the title Pb-based coordination polymer, [Pb_2_(C_24_H_16_N_2_O_8_)(H_2_O)_2_]_*n*_, consists of one Pb^II^ cation, half of a 4,4′-(1,4-phenyl­ene)bis­(2,6-dimethyl­pyridine-3,5-di­carb­oxyl­ate (*L* ^4−^) ligand and one coordinating water mol­ecule. The centers of the benzene ring of the ligand and the four-membered Pb/O/Pb/O ring are located on centers of inversion. The Pb^II^ ion is coordinated in form of a distorted polyhedron by seven O atoms from four separate *L* ^4−^ ligands and by one water O atom. The PbO_7_ polyhedra share O atoms, forming infinite zigzag [PbO_4_(H_2_O)]_*n*_ chains along [100] that are bridged by *L* ^4−^ ligands, forming a two-dimensional coordination network parallel to (001). O—H⋯O hydrogen bonds involving the water mol­ecule are observed.

## Related literature

For background to metal-organic frameworks, see: Long & Yaghi (2009 ▶); Zhao *et al.* (2003 ▶). For related structures, see: Liu *et al.* (2002 ▶); O’Keeffe *et al.* (2008 ▶); Zhang *et al.* (2011 ▶). For lead complexes, see: Harrowfield *et al.* (2004 ▶); Yang *et al.* (2007 ▶). For typical Pb—O distances, see: Chen *et al.* (2012 ▶); Wei *et al.* (2005 ▶). For the photoluminescent mechanism of ligand–metal charge transfer, see: Hu *et al.* (2010 ▶); Zhang *et al.* (2012 ▶). 
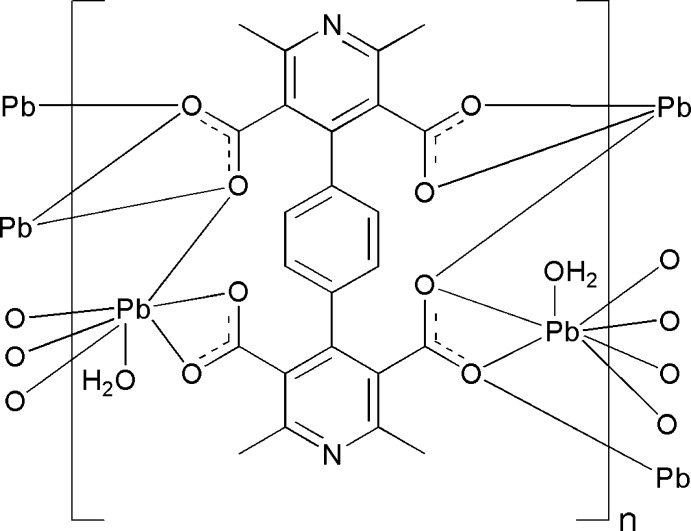


## Experimental

### 

#### Crystal data

[Pb_2_(C_24_H_16_N_2_O_8_)(H_2_O)_2_]
*M* *_r_* = 910.80
Triclinic, 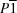

*a* = 7.2182 (12) Å
*b* = 9.0635 (14) Å
*c* = 9.9589 (15) Å
α = 79.202 (2)°
β = 71.683 (2)°
γ = 85.494 (3)°
*V* = 607.43 (17) Å^3^
*Z* = 1
Mo *K*α radiation
μ = 13.90 mm^−1^
*T* = 298 K
0.25 × 0.23 × 0.23 mm

#### Data collection

Bruker SMART APEXII CCD diffractometer
Absorption correction: multi-scan (*SADABS*; Sheldrick, 2003 ▶) *T*_min_ = 0.129, *T*_max_ = 0.142
3168 measured reflections
2119 independent reflections
1932 reflections with *I* > 2σ(*I*)
*R*_int_ = 0.015

#### Refinement

*R*[*F* ^2^ > 2σ(*F* ^2^)] = 0.022
*wR*(*F* ^2^) = 0.055
*S* = 1.01
2119 reflections
174 parameters
H-atom parameters constrained
Δρ_max_ = 1.15 e Å^−3^
Δρ_min_ = −1.16 e Å^−3^

### 

Data collection: *APEX2* (Bruker, 2010 ▶); cell refinement: *SAINT-Plus* (Bruker, 2008) ▶; data reduction: *SAINT-Plus*; program(s) used to solve structure: *SHELXS97* (Sheldrick, 2008 ▶); program(s) used to refine structure: *SHELXL97* (Sheldrick, 2008 ▶); molecular graphics: *SHELXTL* (Sheldrick, 2008 ▶); software used to prepare material for publication: *PLATON* (Spek, 2009 ▶).

## Supplementary Material

Crystal structure: contains datablock(s) I, global. DOI: 10.1107/S1600536813007733/im2422sup1.cif

Structure factors: contains datablock(s) I. DOI: 10.1107/S1600536813007733/im2422Isup2.hkl

Additional supplementary materials:  crystallographic information; 3D view; checkCIF report

## Figures and Tables

**Table 1:** Hydrogen-bond geometry (Å, °)

*D*—H⋯*A*	*D*—H	H⋯*A*	*D*⋯*A*	*D*—H⋯*A*
O5—H2⋯O4^i^	0.85	2.04	2.834 (6)	155
O5—H1⋯O3^ii^	0.85	2.05	2.879 (5)	165

Symmetry codes: (i) 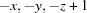; (ii) 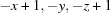.

**Fig. 1.**
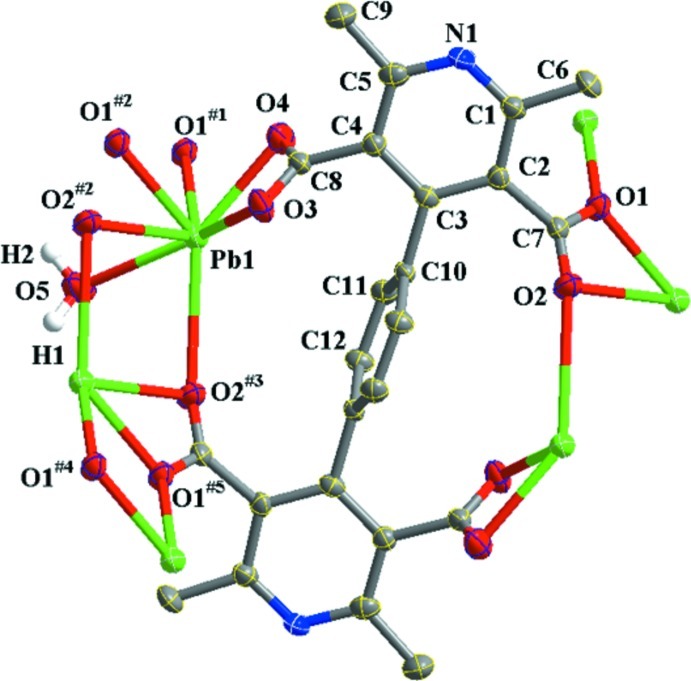
Part of crystal structure of the L4- ligand and PbII centres in 1. All H atoms have been omitted for clarity. [symmetry code: (#1) -x, y+1, -z+1; (#2) x, y - 1,z; (#3) -x + 1, -y + 1, -z + 1; (#4) x + 1, y-1, z; (#5) -x + 1, -y + 1, -z + 1.]

**Fig. 2.**
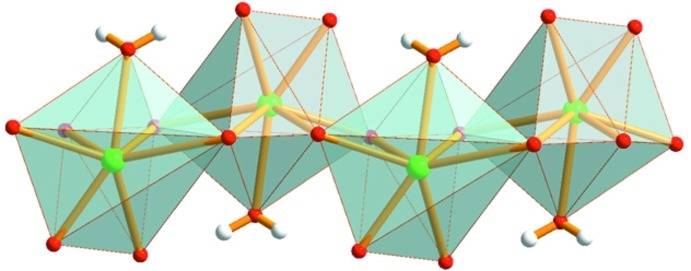
Sectional crystal structure of zigzag chain [PbO4(H2O)]n. Pb, green; O, red; H, white

**Fig. 3.**
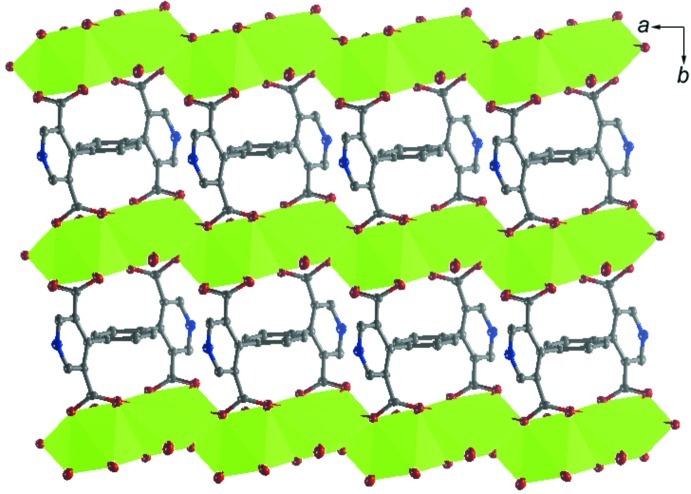
View along c axis of two-dimensional coordination polymer from Pb ions and L4- ligands. PbO7, polyhedron: green; O: red; N: blue; C: grey.

**Fig. 4.**
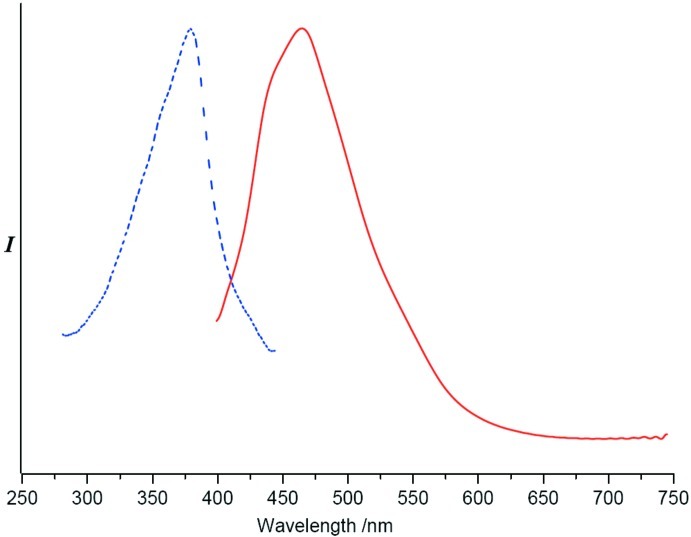
Photoluminescent spectra of 1 (λem at 465 nm, upon λex at 380 nm). I = relative intensity, em = emission, and ex = excitation.

## supplementary crystallographic information

### Comment

In recent years, the chemistry of novel metal-organic hybrid coordination polymers has been the subject of intensive research, due to their interesting molecular structures and their potential as a new class of solid-state materials applied in catalysis, molecular recognition, gas storage, drug delivery, and so on (Liu *et al.*, 2002; O'Keeffe *et al.*, 2008). Generally speaking, the diversity of potential applications in the framework structures of such materials greatly depends on the selection of the metal centers and organic spacers. Recently, carboxylate groups are frequently exploited in the design, syntheses, and crystallization of coordination frameworks, because they exhibit diverse coordination modes, which can enhance the robustness of the architectures. Furthermore, the flexibility of carboxylate groups is always efficient to form fascinating structures. In this paper, we choose a new flexible and multidentate carboxylate ligand, 4,4'-(1,4-phenylene)bis(2,6-dimethylpyridine-3,5-dicarboxylic acid) (H_4_L).

Up to date, research on coordination polymers has focused on transition metal ions as coordination centers, while less concentration has been given to heavy *p*-block metal ion, e.g. lead(II). In contrast to transiton metal ions, lead(II), with its large radius, flexible coordination environment, and variable stereochemical activity, provides unique opportunities for the formation of unusual structures with interesting properties (Harrowfield *et al.*., 2004; Yang *et al.*., 2007). In addition, the intrinsic features of lead(II), the presence of a 6 s^2^ outer electron configuration, inspire chemists extensive interest in coordination chemistry, photophysics, and photochemistry. Herein, we report a new photoluminescent complex [Pb(*L*)(H_2_O)]_n_(1) from the flexible 4,4'-(1,4-phenylene)bis(2,6-dimethylpyridine-3,5- dicarboxylic acid) (H_4_L) and lead salt.

X-ray diffraction analyses reveal that each asymmetric unit of 1 contains half deprotonated *L*^4-^ ligand, one H_2_O molecule and one crystallographically independent Pb^II^ center(Fig 1). Pb1 center is coordinated with seven O atoms: six (O1^#1^, O1^#2^, O2^#2^,O2^#3^, O3, O4) from four H_4_L ligands and one (O5) from the H_2_O molecule. Of particular interest is the weak coordinative bond that exists between Pb1 and O2^#3^. Pb1,O1^#1^, O1^#2^, O2^#2^,O2^#3^, O3, O4, O5 furnish a polyhydral coordination environment (PbO_7_) with the Pb—O bond lengths are in agreement with those reported in other Pb(II) complexes of O-chelating ligands (Wei *et al.*, 2005; Chen *et al.*, 2012).

As shown in Fig.1, each H_4_L ligand connects six crystallographically equivalent Pb atoms. The carboxylato group with O1 and O2 coordinates three lead atoms producing two Pb_2_O_2_ rings that share one common lead atom. The other carboxylate moiety with donor atoms O3 and O4 coordinates one lead atom in a chelating mode. Notably, the resulting PbO_7_ polyhedra share the O1^#4^, O1^#5^, O2^#2^, O2^#3^ atoms to form infinite zigzag chains composed of [PbO_4_(H_2_O)]_n_ in which adjacent Pb atoms are coplanar and Pb···Pb distances are 4.077 Å and 4.161 Å respectively (Fig. 2). Another interesting structural feature of complex 1 is that the zigzag [PbO_4_(H_2_O)]_n_ chains are bridged by H_4_L ligands to form a two-dimensional (2-D) coordination network (Fig. 3).

The photoluminescence spectrum of compound 1 was measured in the solid state at room temperature, as shown in Fig. 4. At room temperature the photoluminescent emission maximum of free H_4_L was observed at 426 nm (upon *λ*_Ex, max_ = 208 nm). For compound 1, excitation at 380 nm leads to strong photoluminescence with an emission maximum at *λ* = 465 nm. The emission peak of complex 1 is red-shifted by about 40 nm compared to that of the pure H_4_L ligand, which can be assigned to the ligand-metal charge transfer (LMCT) (Hu *et al.*, 2010; Zhang *et al.*, 2011; Zhang *et al.*, 2012).

### Experimental

A mixture of Pb(NO_3_)_2_ × 6 H_2_O (66 mg), H_4_L (40 mg) and DMF (6 ml) was sealed in a 25 ml Teflon-lined stainless steel reactor. The mixture was heated to 373 K for 3 days and then cooled to room temperature. The crystal samples were washed with methanol to yield 18 mg of compound 1.

### Refinement

Methyl H atoms were constrained to an ideal geometry (C—H = 0.96 Å), with *U*_iso_(H) =1.5*U*_eq_(C), but were allowed to rotate freely. Other H atoms attached to C atoms were refined using a riding model [C—H = 0.93 Å (CH) and *U*_iso_(H) = 1.2*U*_eq_ (parent atom)].

### Crystal data

**Table:**

[Pb_2_(C_24_H_16_N_2_O_8_)(H_2_O)_2_]	*Z* = 1
*M**_r_* = 910.80	*F*(000) = 422
Triclinic, *P*1	*D*_x_ = 2.490 Mg m^−^^3^
Hall symbol: -P 1	Mo *K*α radiation, λ = 0.71073 Å
*a* = 7.2182 (12) Å	Cell parameters from 1580 reflections
*b* = 9.0635 (14) Å	θ = 2.7–23.1°
*c* = 9.9589 (15) Å	µ = 13.90 mm^−^^1^
α = 79.202 (2)°	*T* = 298 K
β = 71.683 (2)°	Block, colorless
γ = 85.494 (3)°	0.25 × 0.23 × 0.23 mm
*V* = 607.43 (17) Å^3^	

### Data collection

**Table:**

Bruker SMART APEXII CCD diffractometer	2119 independent reflections
Radiation source: fine-focus sealed tube	1932 reflections with *I* > 2σ(*I*)
Graphite monochromator	*R*_int_ = 0.015
phi and ω scans	θ_max_ = 25.0°, θ_min_ = 2.2°
Absorption correction: multi-scan (*SADABS*; Sheldrick, 2003)	*h* = −8→8
*T*_min_ = 0.129, *T*_max_ = 0.142	*k* = −10→10
3168 measured reflections	*l* = −10→11

### Refinement

**Table:**

Refinement on *F*^2^	Primary atom site location: structure-invariant direct methods
Least-squares matrix: full	Secondary atom site location: difference Fourier map
*R*[*F*^2^ > 2σ(*F*^2^)] = 0.022	Hydrogen site location: inferred from neighbouring sites
*wR*(*F*^2^) = 0.055	H-atom parameters constrained
*S* = 1.01	*w* = 1/[σ^2^(*F*_o_^2^) + (0.0347*P*)^2^] where *P* = (*F*_o_^2^ + 2*F*_c_^2^)/3
2119 reflections	(Δ/σ)_max_ < 0.001
174 parameters	Δρ_max_ = 1.15 e Å^−^^3^
0 restraints	Δρ_min_ = −1.16 e Å^−^^3^

### Special details

**Table:**

Geometry. All e.s.d.'s (except the e.s.d. in the dihedral angle between two l.s. planes) are estimated using the full covariance matrix. The cell e.s.d.'s are taken into account individually in the estimation of e.s.d.'s in distances, angles and torsion angles; correlations between e.s.d.'s in cell parameters are only used when they are defined by crystal symmetry. An approximate (isotropic) treatment of cell e.s.d.'s is used for estimating e.s.d.'s involving l.s. planes.
Refinement. Refinement of *F*^2^ against ALL reflections. The weighted *R*-factor *wR* and goodness of fit *S* are based on *F*^2^, conventional *R*-factors *R* are based on *F*, with *F* set to zero for negative *F*^2^. The threshold expression of *F*^2^ > σ(*F*^2^) is used only for calculating *R*-factors(gt) *etc*. and is not relevant to the choice of reflections for refinement. *R*-factors based on *F*^2^ are statistically about twice as large as those based on *F*, and *R*- factors based on ALL data will be even larger.

### Fractional atomic coordinates and isotropic or equivalent isotropic displacement parameters (Å^2^)

**Table:**

	*x*	*y*	*z*	*U*_iso_*/*U*_eq_	
Pb1	0.24557 (3)	0.11141 (2)	0.47717 (2)	0.02892 (9)	
O1	0.0452 (5)	0.9117 (4)	0.6086 (4)	0.0281 (8)	
O2	0.3224 (5)	0.8538 (4)	0.6568 (5)	0.0365 (9)	
O3	0.2253 (5)	0.1995 (4)	0.7194 (4)	0.0343 (9)	
O4	−0.0248 (6)	0.2448 (5)	0.6332 (5)	0.0382 (10)	
O5	0.3782 (6)	−0.0957 (5)	0.3132 (5)	0.0441 (11)	
H1	0.4881	−0.1412	0.3019	0.066*	
H2	0.2924	−0.1629	0.3379	0.066*	
N1	−0.2153 (6)	0.5547 (5)	0.9379 (5)	0.0275 (10)	
C1	−0.1131 (7)	0.6798 (6)	0.8704 (6)	0.0245 (11)	
C2	0.0591 (7)	0.6774 (5)	0.7553 (5)	0.0220 (11)	
C3	0.1282 (7)	0.5404 (6)	0.7125 (5)	0.0213 (10)	
C4	0.0148 (7)	0.4140 (6)	0.7758 (5)	0.0233 (11)	
C5	−0.1579 (7)	0.4255 (6)	0.8888 (6)	0.0271 (12)	
C6	−0.1922 (8)	0.8214 (6)	0.9291 (6)	0.0341 (13)	
H6A	−0.2907	0.8662	0.8872	0.051*	
H6B	−0.0882	0.8906	0.9060	0.051*	
H6C	−0.2481	0.7975	1.0315	0.051*	
C7	0.1542 (7)	0.8222 (6)	0.6705 (6)	0.0242 (11)	
C8	0.0752 (7)	0.2736 (6)	0.7103 (6)	0.0243 (11)	
C9	−0.2867 (8)	0.2946 (7)	0.9630 (7)	0.0379 (14)	
H9A	−0.2869	0.2692	1.0611	0.057*	
H9B	−0.2388	0.2102	0.9154	0.057*	
H9C	−0.4172	0.3203	0.9603	0.057*	
C10	0.3206 (7)	0.5253 (5)	0.5999 (5)	0.0192 (10)	
C11	0.3313 (7)	0.5098 (6)	0.4608 (5)	0.0249 (11)	
H11	0.2177	0.5160	0.4347	0.030*	
C12	0.5090 (7)	0.4854 (6)	0.3615 (5)	0.0241 (11)	
H12	0.5146	0.4761	0.2689	0.029*	

### Atomic displacement parameters (Å^2^)

**Table:**

	*U*^11^	*U*^22^	*U*^33^	*U*^12^	*U*^13^	*U*^23^
Pb1	0.02297 (13)	0.02526 (13)	0.03649 (14)	−0.00185 (8)	−0.00454 (9)	−0.00765 (9)
O1	0.0269 (19)	0.0225 (19)	0.037 (2)	−0.0014 (15)	−0.0143 (17)	−0.0010 (16)
O2	0.024 (2)	0.029 (2)	0.054 (3)	−0.0052 (16)	−0.0106 (19)	−0.0020 (18)
O3	0.034 (2)	0.029 (2)	0.044 (2)	0.0113 (17)	−0.0165 (19)	−0.0119 (18)
O4	0.030 (2)	0.036 (2)	0.056 (3)	0.0017 (17)	−0.017 (2)	−0.022 (2)
O5	0.029 (2)	0.048 (3)	0.059 (3)	0.0011 (19)	−0.013 (2)	−0.021 (2)
N1	0.021 (2)	0.031 (3)	0.026 (2)	0.0005 (19)	0.0000 (19)	−0.008 (2)
C1	0.020 (3)	0.027 (3)	0.026 (3)	0.002 (2)	−0.006 (2)	−0.008 (2)
C2	0.021 (2)	0.018 (3)	0.027 (3)	0.002 (2)	−0.009 (2)	−0.004 (2)
C3	0.020 (2)	0.026 (3)	0.018 (2)	0.002 (2)	−0.006 (2)	−0.005 (2)
C4	0.023 (3)	0.022 (3)	0.024 (3)	0.001 (2)	−0.007 (2)	−0.005 (2)
C5	0.021 (3)	0.033 (3)	0.027 (3)	−0.001 (2)	−0.009 (2)	−0.002 (2)
C6	0.031 (3)	0.029 (3)	0.037 (3)	0.006 (2)	0.000 (3)	−0.014 (2)
C7	0.024 (3)	0.019 (3)	0.027 (3)	0.001 (2)	−0.001 (2)	−0.007 (2)
C8	0.020 (3)	0.021 (3)	0.027 (3)	0.002 (2)	−0.002 (2)	−0.003 (2)
C9	0.030 (3)	0.034 (3)	0.041 (4)	−0.006 (3)	0.001 (3)	−0.003 (3)
C10	0.018 (2)	0.017 (2)	0.020 (2)	0.0026 (19)	−0.003 (2)	−0.0028 (19)
C11	0.020 (2)	0.028 (3)	0.029 (3)	0.001 (2)	−0.011 (2)	−0.005 (2)
C12	0.023 (3)	0.030 (3)	0.019 (3)	0.001 (2)	−0.005 (2)	−0.004 (2)

### Geometric parameters (Å, º)

**Table:**

Pb1—O1^i^	2.327 (4)	C2—C3	1.393 (7)
Pb1—O4	2.472 (4)	C2—C7	1.507 (7)
Pb1—O1^ii^	2.538 (3)	C3—C4	1.390 (7)
Pb1—O3	2.638 (4)	C3—C10	1.501 (7)
Pb1—O5	2.644 (4)	C4—C5	1.405 (7)
O3—C8	1.248 (6)	C5—C9	1.494 (8)
O4—C8	1.277 (7)	C6—H6A	0.9600
C8—C4	1.510 (7)	C6—H6B	0.9600
O1—C7	1.294 (6)	C6—H6C	0.9600
O1—Pb1^iii^	2.327 (4)	C9—H9A	0.9600
O1—Pb1^ii^	2.538 (3)	C9—H9B	0.9600
N1—C5	1.338 (7)	C9—H9C	0.9600
N1—C1	1.348 (7)	C10—C12^iv^	1.391 (7)
O2—C7	1.230 (6)	C10—C11	1.395 (7)
O5—H1	0.8500	C11—C12	1.383 (7)
O5—H2	0.8500	C11—H11	0.9300
C1—C2	1.403 (7)	C12—C10^iv^	1.391 (7)
C1—C6	1.507 (7)	C12—H12	0.9300
			
O1^i^—Pb1—O4	79.28 (13)	C3—C4—C5	119.1 (5)
O1^i^—Pb1—O1^ii^	66.21 (14)	C3—C4—C8	117.7 (4)
O4—Pb1—O1^ii^	75.30 (12)	C5—C4—C8	122.9 (5)
O1^i^—Pb1—O3	89.30 (13)	N1—C5—C4	121.7 (5)
O4—Pb1—O3	51.16 (12)	N1—C5—C9	116.0 (5)
O1^ii^—Pb1—O3	124.84 (11)	C4—C5—C9	122.3 (5)
O1^i^—Pb1—O5	78.92 (13)	C1—C6—H6A	109.5
O4—Pb1—O5	151.48 (13)	C1—C6—H6B	109.5
O1^ii^—Pb1—O5	79.19 (12)	H6A—C6—H6B	109.5
O3—Pb1—O5	146.08 (13)	C1—C6—H6C	109.5
C8—O3—Pb1	87.8 (3)	H6A—C6—H6C	109.5
C8—O4—Pb1	94.7 (3)	H6B—C6—H6C	109.5
O3—C8—O4	122.3 (5)	O2—C7—O1	121.6 (5)
O3—C8—C4	121.6 (5)	O2—C7—C2	124.0 (5)
O4—C8—C4	115.8 (4)	O1—C7—C2	114.4 (4)
C7—O1—Pb1^iii^	104.5 (3)	C5—C9—H9A	109.5
C7—O1—Pb1^ii^	136.9 (3)	C5—C9—H9B	109.5
Pb1^iii^—O1—Pb1^ii^	113.79 (14)	H9A—C9—H9B	109.5
C5—N1—C1	119.3 (4)	C5—C9—H9C	109.5
Pb1—O5—H1	125.1	H9A—C9—H9C	109.5
Pb1—O5—H2	107.8	H9B—C9—H9C	109.5
H1—O5—H2	106.8	C12^iv^—C10—C11	119.3 (4)
N1—C1—C2	122.0 (5)	C12^iv^—C10—C3	118.9 (4)
N1—C1—C6	115.8 (5)	C11—C10—C3	121.6 (4)
C2—C1—C6	122.2 (5)	C12—C11—C10	120.7 (5)
C3—C2—C1	118.6 (5)	C12—C11—H11	119.7
C3—C2—C7	120.8 (5)	C10—C11—H11	119.7
C1—C2—C7	120.2 (4)	C11—C12—C10^iv^	120.0 (5)
C4—C3—C2	118.8 (5)	C11—C12—H12	120.0
C4—C3—C10	119.1 (4)	C10^iv^—C12—H12	120.0
C2—C3—C10	122.1 (5)		

Symmetry codes: (i) *x*, *y*−1, *z*; (ii) −*x*, −*y*+1, −*z*+1; (iii) *x*, *y*+1, *z*; (iv) −*x*+1, −*y*+1, −*z*+1.

### Hydrogen-bond geometry (Å, º)

**Table:**

*D*—H···*A*	*D*—H	H···*A*	*D*···*A*	*D*—H···*A*
O5—H2···O4^v^	0.85	2.04	2.834 (6)	155
O5—H1···O3^vi^	0.85	2.05	2.879 (5)	165

Symmetry codes: (v) −*x*, −*y*, −*z*+1; (vi) −*x*+1, −*y*, −*z*+1.
